# Linc00996 is a favorable prognostic factor in LUAD: Results from bioinformatics analysis and experimental validation

**DOI:** 10.3389/fgene.2022.932973

**Published:** 2022-09-02

**Authors:** Zhenghai Shen, Xin Li, Zaoxiu Hu, Yanlong Yang, Zhenghong Yang, Shanshan Li, Yongchun Zhou, Jie Ma, Hongsheng Li, Xi Liu, Jingjing Cai, Lisa Pu, Xiaoxiong Wang, Yunchao Huang

**Affiliations:** ^1^ Cancer Center Office, The Third Affiliated Hospital of Kunming Medical University (Yunnan Cancer Hospital, Yunnan Cancer Center), Kunming, China; ^2^ Molecular Diagnosis Sub Center of Yunnan Cancer Center, Yunnan Cancer Molecular Diagnosis Center, The Third Affiliated Hospital of Kunming Medical University (Yunnan Cancer Hospital, Yunnan Cancer Center), Kunming, China; ^3^ Department of Thoracic Surgery I, The Third Affiliated Hospital of Kunming Medical University (Yunnan Cancer Hospital, Yunnan Cancer Center), Kunming, China; ^4^ Department of Clinical Laboratory, The Third Affiliated Hospital of Kunming Medical University (Yunnan Cancer Hospital, Yunnan Cancer Center), Kunming, China; ^5^ Department of Pathology, The Third Affiliated Hospital of Kunming Medical University (Yunnan Cancer Hospital, Yunnan Cancer Center), Kunming, China; ^6^ Department of Thoracic Surgery, The First Affiliated Hospital of Kunming Medical University, Kunming, China; ^7^ Department of Nephrology, Kunming Yanan Hospital, Kunming, China

**Keywords:** Linc00996, lncRNA, LUAD, bioinformatics, prognosis

## Abstract

**Background:** Linc00996 has been reported in a variety of malignant tumors, but its potential role and significance in lung adenocarcinoma (LUAD) are not fully understood. The authors investigated the expression and biological behavior of Linc00996 in LUAD and elucidated the function of its potential target genes.

**Materials and methods:** The data of Linc00996 expression in cancers were derived from GEPIA. GEO and TCGA datasets were used to identify the differential expression of Linc00996 in LUAD and analyze the respective correlation between different expression levels and LUAD stage and survival prognosis. We further elucidated the potential biological processes and pathways involved with Linc00996 in LAUD by GSEA. ssGSEA was applied to explore the relationship between Linc00996 and immune activity. Finally, the clinical impact of Linc00996 was assessed in 61 patients with LUAD, and the biological functions of Linc00996 were determined by a series of experiments *in vitro*, such as CCK8, colony formation, migration, and invasion assays.

**Results:** Compared with adjacent normal lung tissues, Linc00996 was significantly downregulated in LUAD, and its expression was negatively correlated with T stage, N stage, and pathological stage. An *in vitro* study suggested that enhanced Linc00996 expression could inhibit cell proliferation, clonal formation, migration, and invasion in LUAD cell lines. Via GSEA and ssGSEA, we observed that Linc00996 might be connected with immune infiltration in LUAD, and Linc00996 might inhibit tumorigenesis and metastasis by regulating antigen processing and presentation, JAK-STAT3, and cell adhesion molecular signaling pathways.

**Conclusion:** Linc00996 is a novel tumor suppressor in LUAD and may suppress the tumorigenesis and metastasis of LUAD via the tumor-related signaling pathway, such as antigen processing and presentation, JAK-STAT3, and cell adhesion molecular signaling pathways.

## Introduction

Cancer is a major disease worldwide and seriously threatens human health. There were more than 18 million new cancer cases and about 9.6 million deaths worldwide in 2018, and almost half of the new cases and over half of the cancer deaths worldwide occurred in Asia in 2018, especially in China, the burden of cancer is growing, and approximately 22% of all new cases worldwide occur in China (Bray et al., 2018). In recent years, the incidence and mortality of lung cancer have shown a rising trend, with non-small-cell lung cancer (NSCLC) accounting for more than 80% (Ettinger et al., 2013; Siegel et al., 2021). Lung adenocarcinoma (LUAD) has gradually increased in the proportion of non-small-cell lung cancer and is the most common subtype of NSCLC (Kadara et al., 2017). LUAD patients have been shown to have a shorter survival time than other NSCLC patients (Youlden et al., 2008). Surgical resection is the primary treatment for curative outcomes for LUAD. However, the postoperative recurrence rate of LUAD is as high as 35%–50%, and the prognosis remains poor. Although a series of advanced therapies, such as targeted therapy and immunotherapy, have greatly increased the overall survival of lung cancer patients, the 5-year overall survival rate of these patients remains less than 20% (Alam et al., 2020). Therefore, the search for new prognostic biomarkers is urgent.

Non-coding RNAs (ncRNAs) refer to RNA molecules that are transcribed from the genome and do not encode proteins play an important role in gene activation and silencing, transcriptional regulation, and RNA splicing and modification (Liu et al., 2019). Long non-coding RNAs (lncRNAs) are important products of non-coding genes and regulate gene expression at both transcriptional and post-transcriptional levels (Ramanathan et al., 2019). LncRNAs are closely related to diseases, especially tumors, among which Linc00996 can be used as a prognostic factor for colorectal cancer (Ge et al., 2018). Based on Linc00996 construct, a risk score system was established to predict survival for multiple myeloma and head and neck squamous cell carcinoma (HNSCC) (Zhou et al., 2020; Lina, 2021). Previous analysis found that Linc00996 was downregulated in LUAD; the RNA-seq analysis based on A549 with overexpressed Linc00996 indicated that differentially expressed genes (DEGs) were closely related to the immune pathway and were enriched in immune gene sets (Yan et al., 2021). However, its specific role in LUAD has not been clearly defined. Therefore, our study systematically explored the role of the Linc00996 gene in LUAD based on bioinformatics, tissue sample validation, and cell function experiments.

## Materials and methods

### Data collection

The expression matrix data and survival status information of 59 normal tissue samples and 535 cancer tissue samples were downloaded from TCGA database (https://portal.gdc.cancer.gov) (Release Date: 23/08/2018; Release Number: 12.0). When removing duplicate samples and screening 11A, 01A, and 02A samples, we obtained 58 normal samples and 504 cancer samples to compare the expression levels of normal and cancer samples. After 163 samples lacking survival information were removed, 341 cancer samples were used for survival analysis. The relationship between Linc00996 and clinicopathological features and prognosis was evaluated in 331 cancer samples with sufficient clinical information. GSE31210 (226 tumor samples and 20 normal samples in total) and GSE37745 (106 LUAD tumor samples) from GEO (https://www.ncbi.nlm.nih.gov/geo/) were also used to evaluate the clinical and prognostic significance of Linc00996 in NSCLC.

### Feature-rich analysis

DEGs were analyzed for their Gene Ontology (GO) function and Kyoto Encyclopedia of Genes and Genomes (KEGG) pathway enrichment using the R package “poly-profiler.” Additionally, the R package “ggplot2” was used for demonstration.

### Protein–Protein interaction

The differentially expressed genes with a strong correlation with Linc00996 (Pearson correlation analysis, |Cor|>0.6) were used to predict the PPI interaction network using the STRING database (https://string-db.org/cgi/network.pl), and Cytoscape was used to visualize the network.

### Construction of prognostic gene signatures

The R package “glmnet” was applied for LASSO Cox analysis. “Cox” was the family parameter, and genes authenticated in the LASSO analysis were placed as covariates and included in the multivariate Cox regression analysis. Finally, prognostic gene signatures were set up on account of expression and regression coefficients. Throughout the whole data analysis process, LASSO can filter combinations of independent variables and obtain better fits by adding constraints to reduce the dimensionality of high-dimensional data.

### Tissue specimens

This study was carried out with the approval of the Medical Ethics Committee of The Third Affiliated Hospital of Kunming Medical University, and informed consent was obtained before surgery. A total of 61 freshly frozen human NSCLC tissues were collected.

### RNA purification and cDNA synthesis

According to the manufacturer’s instructions, fresh-frozen tissues were sufficiently minced in lysis buffer, and total RNA was extracted using an RNA-Quick Purification Kit (ES Science, China). RNA was dissolved in 25 µL of elution buffer (ES Science, China), and RNA concentration and purity were determined using a NanoDrop spectrophotometer (Thermo, United States). A measure of 1 g of RNA was DNase-treated and reverse transcribed into 20 µL cDNA (Vazyme, China).

### Quantitative real-time PCR

cDNA was diluted to a concentration of 1:2 and analyzed using a 7500 Fast Real-Time PCR system (Thermo, United States) and detected using SYBR Green I. In a 96-well plate (Nest, Germany), the reaction mixture containing Taq Pro Universal SYBR qPCR Master Mix and primers (final volume was 20 µL) was prepared, and each sample was replicated three times. The primer sequences of Linc00996 and GAPDH are listed in Supplementary Table S1.

### Cell cultures

A549 and NCI-H1734 cells were obtained from the National Collection of Authenticated Cell Cultures of China, and both belonged to human lung adenocarcinoma cell Lines. Two cell lines were cultured in DMEM (Gibco, United States) and 10% FBS (Gibco, United States) in an incubator at 37°C and 5% CO_2_.

### Lentivirus and plasmid transfection

The lentiviral vector encoding human Linc00996 was obtained from GenePharma (Shanghai, China). A549 and NCI-H1734 cells were infected with appropriate concentrations of lentivirus, screened with puromycin for 2 weeks, and gene expression was verified by qPCR. The sequence of the plasmid is listed in Supplementary Table S2.

### CCK‐8 and colony formation assays

For the cell counting kit-8 (CCK-8) (GlpBio, China) assay, cells (2×10^3^ per well) were inoculated in 96-well plates and cultured at 37°C. They were tested at the same time every day until 96 h. A measure of 10 μL CCK-8 reagent was added to each well and mixed with a gentle vibration. Then, the cells were incubated at 37°C for 1 h, and the optical density (OD) was measured at 450 nm. For the colony formation assay, cells (500 per well) were inoculated in six-well plates and cultured at 37°C for 10 days. Furthermore, the cell colonies were fixed with 4% paraformaldehyde overnight, dyed with crystal violet, and counted.

### Cell migration and invasion and scratch wound assays

Before the invasion experiment, Matrigel (Corning, NY, United States) was diluted to 1:8 with the culture medium, and 65 µL was added to the upper chamber of each chamber (Falcon, Corning, NY, United States) and incubated at 37°C for 1 h. A549 and NCI-H1734 cells were resuspended in the serum-free medium and added to the upper chamber, respectively (4×10^4^/200 µL for migration assay and 1×10^5^/200 µL for invasion assay). Furthermore, 800 µL culture medium (DMEM containing 20% FBS) was added to the lower chamber and incubated at 37°C for 24 h. Chambers were fixed with 4% paraformaldehyde overnight, dyed with crystal violet, and counted. For the wound healing assay, A549 and NCI-H1734 cells were inoculated in six-well plates for 24 h, scratched with a 1000-µL sterile pipette tip, and the crawling distance was monitored and recorded at 0, 24, and 48 h, respectively.

### Statistical analysis

SPSS 25.0 software was used for statistical analysis, and graphing was performed using GraphPad Prism 7. The two-tailed Pearson chi-squared test or rank sum test was applied to statistically analyze the relationship between Linc00996 expression and clinicopathological parameters. The survival analysis was performed using the “survival” R package, and Kaplan–Meier survival curves assessed the correlation between Linc00996 expression and overall survival (OS). Cox risk regression analyses were utilized to assess whether Linc00996 was an independent prognostic factor for LUAD. The two-tailed Student’s t-test was employed as appropriate. *p* < 0.05 was considered statistically significant.

## Results

### Expression analysis of Linc00996 in lung adenocarcinoma

**FIGURE 1:**
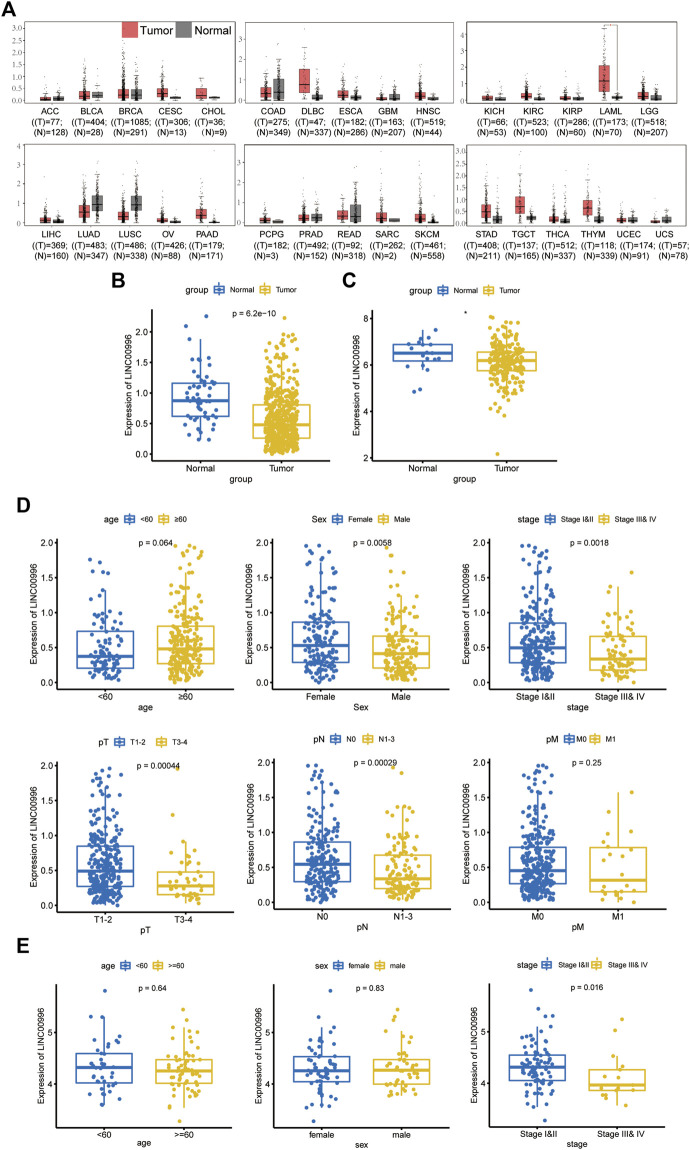
**(A)** GEPIA database analysis of the expression of Linc00996 across cancers. **(B)** Expression of Linc00996 in TCGA-LUAD. **(C)** Expression of Linc00996 in LUAD in GSE31210. **(D,E)** Expression of Linc00996 in TCGA-LUAD and GSE37745 under various clinical traits.

In this study, Linc00996 expression in pan-cancer was construed through the GEPIA database (Figure 1A); TCGA-LUAD and GSE31210 data were normalized by log2, and Linc00996 expression in LUAD was displayed in TCGA-LUAD (Figure 1B) and GSE31210 (Figure 1C). These figures showed that Linc00996 was significantly downregulated in LUAD compared with adjacent normal lung tissues.

### Correlation analysis between Linc00996 expression and clinicopathological data in lung adenocarcinoma

TCGA-LUAD and GSE37745 were applied to analyze the correlation between Linc00996 expression and clinicopathological data in NSCLC. The clinical data of LUAD were divided into different ages (with 60 years old as the cutoff), different sexes, different pathological stages, different T stages, different N stages, and different M stages. In TCGA-LUAD, Linc00996 was significantly underexpressed in males, stages III and IV, T3–4, and N1-3 (Figure 1D). Significantly lower expression of Linc00996 in stages III and IV was also shown in GSE37745 (Figure 1E). According to Linc00996 high- and low-expression groups (taking the median expression of Linc00996 as the cutoff), the correlation between LUAD patients and clinical traits was analyzed (Table 1). The results suggested that Linc00996 expression was negatively correlated with the T stage, N stage, and pathological stage.

**TABLE 1:** Statistics of the expression of Linc00996 in TCGA-LUAD under various clinical traits.

Parameter	Whole cohort (n = 331)	Low Linc00996	High Linc00996	*P*
Age				
≥60	239	112	127	0.064
<60	92	54	38	
Gender				
Female	167	75	92	0.0058
Male	164	91	73	
Pathological stage				
I	166	70	96	0.0018
II	82	43	39	
III	61	41	20	
IV	22	12	10	
T stage				
T1	100	37	63	0.00044
T2	185	96	89	
T3	28	20	8	
T4	18	13	5	
M stage				
M0	309	154	155	0.25
M1	22	12	10	
N stage				
N0	206	88	118	0.00029
N1	71	43	28	
N2	53	35	18	
N3	1	0	1	

### Prognostic analysis of Linc00996 in NSCLC

**FIGURE 2:**
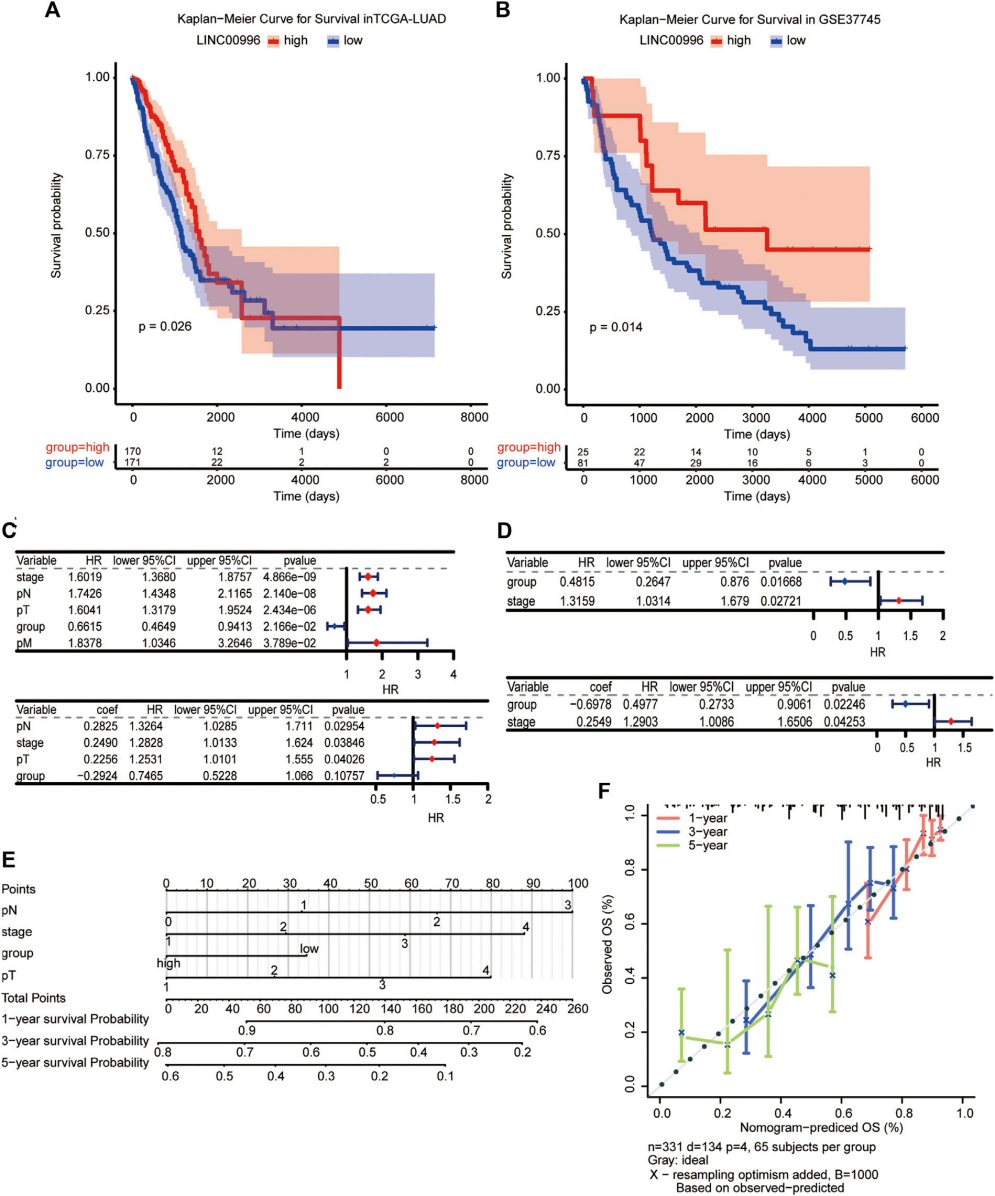
**(A,B)** Kaplan–Meier survival analysis of TCGA-LUAD and GSE37745, with significant differences among different groups, *p* < 0.05. **(C,D)** Independent prognosis forest plot of LUAD patients (TCGA-LUAD univariate and TCGA-LUAD multivariate; GSE37745 univariate and GSE37745 multivariate, respectively). **(E, F)** A visualization model of the nomogram and an overall calibration curve.

To investigate the effects of Linc00996 on survival and prognosis of patients, we used the median value of Linc00996 expression to divide LUAD cancer patients in TCGA-LUAD and GSE37745 into high- and low-Linc00996 groups and analyzed the prognosis and survival between groups. The “survival” R software package was applied to calculate the survival analysis, and the “survminer” package summarized and visualized the results, revealing that the survival time of patients in the low-expression group of Linc00996 was shorter (Figures 2A,B). Univariate and multivariate Cox risk regression analyses were utilized to assess whether Linc00996 was an independent prognostic factor for LUAD in both datasets. In this study, high- and low-expression groups of Linc00996 and clinical traits were used as variables to compare with the survival time; the “survminer” R software package was employed to calculate and predict whether Linc00996 was related to patient survival. In TCGA-LUAD, univariate Cox analysis results were filtered; the threshold was set at *p* < 0.05. Four prognostic-related features were obtained: T, N, stage, and expression of Linc00996. The “forestplot” R package was used to draw univariate and multivariate Cox forest plots, where the outcome event of the study was death. As shown in the results, we found that the three traits T, N, and stage fall on the right side of the invalid line, demonstrating that the incidence rate of the experimental group is greater than that of the control group. In other words, compared with the patients with these three traits, the higher the staging level was, the higher the mortality rate was. Therefore, it can be considered that the three traits are harmful factors for LUAD patients.

Compared with the low-expression group, the high-Linc00996 expression group fell on the left side of the null line, suggesting that the gene is a protective factor. In GSE37745, Linc00996 was a protective factor, and stage was a harmful factor (Figures 2C,D). We drew a visualization model of the nomogram and predicted the possible 1-, 3-, and 5-year survival for LUAD patients (C-index = 0.69) and drew an overall calibration curve to verify that the nomogram predicts better (Figures 2E,F).

### Stratified survival analysis for Linc00996

**FIGURE 3:**
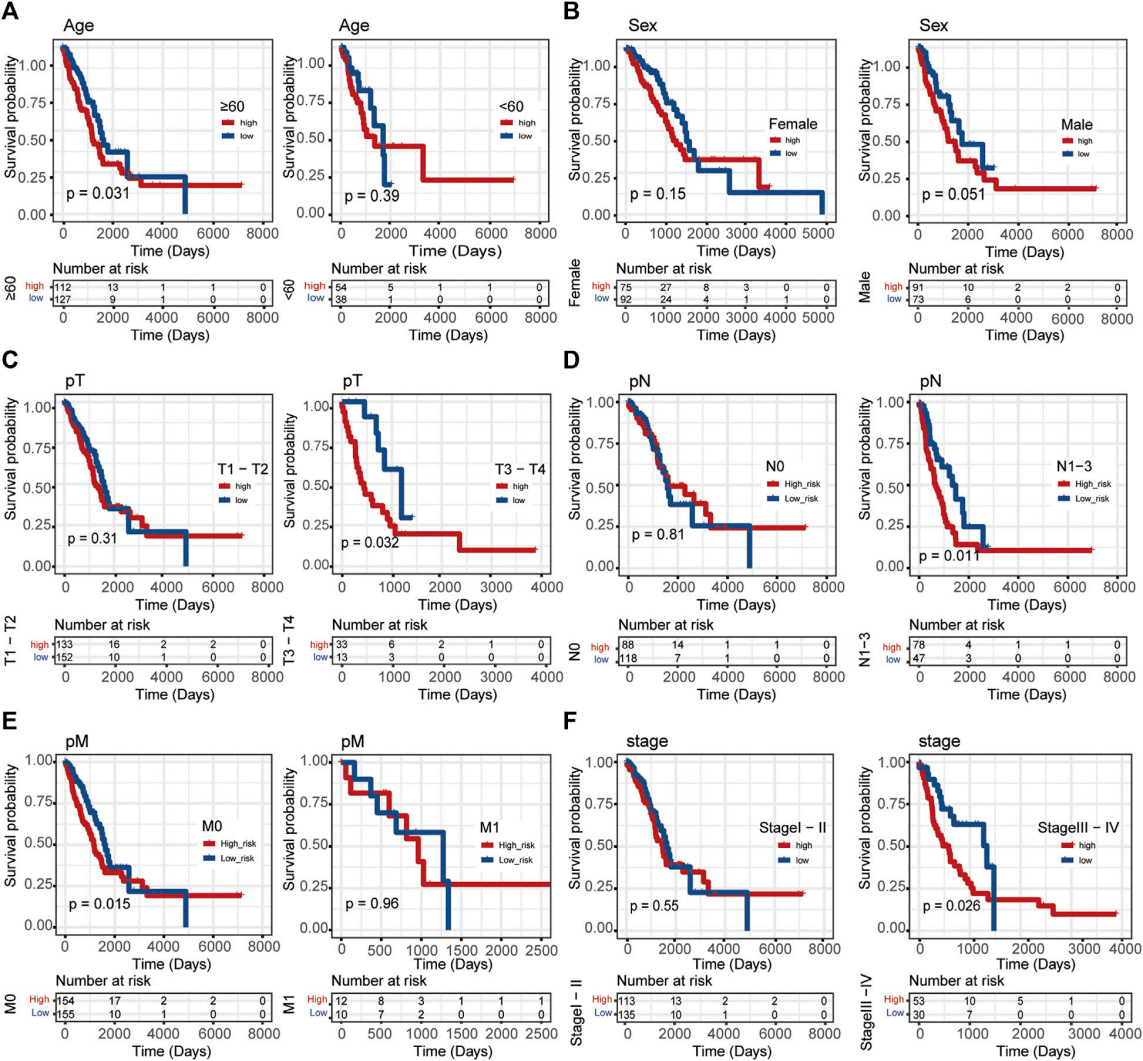
Stratified survival analysis in TCGA-LUAD. **(A)** Age. **(B)** Sex. **(C)** T stage. **(D)** N stage. **(E)** M stage. **(F)** Pathological stage. The ordinate represents the survival rate, the abscissa represents the OS, and the red curve represents the group of samples with high expression of the gene, bounded by the median expression level. The blue curve represents the group of samples with low gene expression, with significant differences among different groups, *p* < 0.05.

In this study, Linc00996 was stratified according to different clinical traits, and it was found that patients with high-Linc00996 expression in M0, N1-3, stages III–IV, T3–4, and ≥60 years had shorter survival, suggesting that high expression of Linc00996 was not conducive to survival (Figure 3).

### Exploration of the potential mechanism by which Linc00996 affects lung adenocarcinoma progression

**FIGURE 4:**
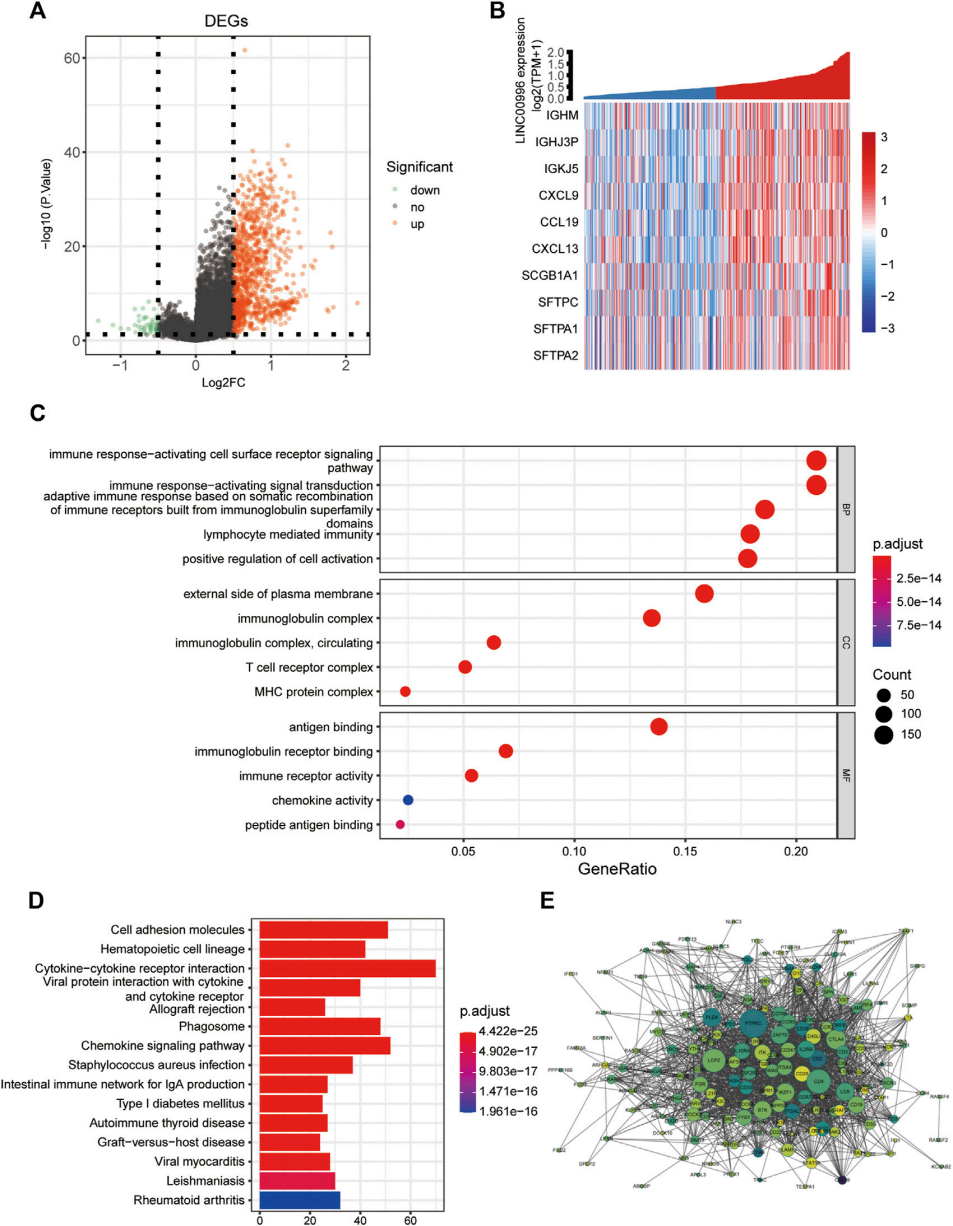
**(A)** Linc00996 high- and low-expression groups, volcano plot of differentially expressed genes. **(B)** Linc00996 high- and low-expression groups, differentially expressed gene heatmap. **(C,D)** Enrichment analysis of DEQSCGs. **(E)** DEG enrichment analysis of the PPI network (color represents logFC, darker indicates larger logFC, and all logFC is greater than 0.5; the size represents the degree of connectivity, and the larger the value is, the higher the degree of connectivity is).

The purpose of differentially expressed gene analysis is to determine the differentially expressed genes (DEGs) between different sample groups and to conduct further in-depth functional mining of their functions. According to the median expression of Linc00996, 165 and 166 samples were included in the high- and low-expression groups, respectively. The “limma” software package was performed to analyze the DEGs between the high- and low-expression groups of Linc00996 in TCGA-LUAD (lncRNA and mRNA expression matrix), and the threshold was *p* < 0.05 and | logFc | >0.5. A total of 1,058 genes were significantly different between two groups, including 1,009 upregulated genes and 49 downregulated genes. Meanwhile, a volcanic map was drawn to display the overall distribution of DEGs, as shown in Figure 4A. The volcano plot and heatmap were drawn using the “ggplot” and “pheatmap” R software packages, respectively (Figure 4B); the correlation coefficient R and statistical discrepancy are listed in Supplementary Table S3.

### Differential gene enrichment analysis

GO analysis and KEGG approach enrichment analysis were carried out based on DEGs; the threshold was set at *p*-value < 0.05. A total of 1,113 GO entries were enriched, including 940 BPs, 76 CCs, and 97 MFs, and 62 KEGG pathways were enriched. As shown in Figure 4C, biological processes were mainly enriched in immune response-activating cell surface receptor signaling pathways, immune response-activating signal transduction, and adaptive immune response based on somatic recombination of immune receptors from immunoglobulin superfamily domains, lymphocyte-mediated immunity, and positive regulation of cell activation, etc. For cellular components, the findings showed that genes were involved in immunoglobulin complexes, outside of the plasma membrane, circulating immunoglobulin complexes, MHC protein complexes, and T-cell receptor complexes, etc. Molecular functions were primarily concentrated in immunoglobulin receptor binding, peptide antigen binding, and chemokine activity, etc. The KEGG pathway, as shown in Figure 4D, was mainly enriched in cell adhesion molecules, hematopoietic cell lineage, allogeneic transplant rejection and chemokine signaling pathway, and intestinal immune network for IgA production, etc. Using the STRING database, the screened differentially expressed genes with a strong correlation with Linc00996 were predicted by the PPI interaction network (Figure 4E).

### Signaling pathways related to Linc00996 expression

**FIGURE 5:**
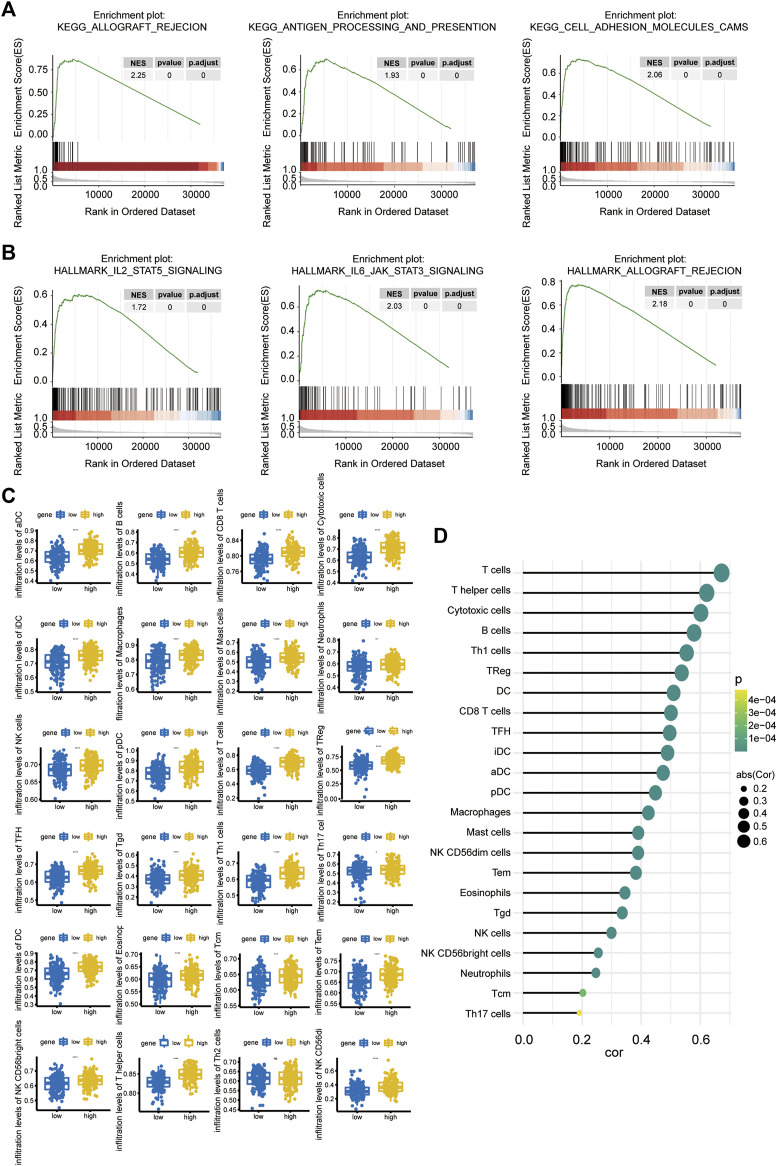
**(A,B)** GSEA of Linc00996 **(**KEGG pathway in the upper row and the hallmark pathway in the lower row). **(C)** Differences among 22 TIICs. The abscissa represents the high- and low-expression groups, and the ordinate represents immune cells. Blue indicates the low-expression group, and yellow indicates the high-expression group. *p < 0.05, **p < 0.01, ***p < 0.001, ****p < 0.0001. **(D)** Correlation between Linc00996 expression and the proportion of significant immune cells.

To further explore the possible related signaling pathways and potential biological functions of Linc00996 in LUAD, we calculated the correlation between Linc00996 and other genes in 331 samples and sorted all genes according to the correlation from high to low. The sorted genes were used as the gene set to be tested, and the KEGG signaling pathway and hallmark were used as the predefined gene set to detect the enrichment of the KEGG signaling pathway and hallmark in the gene set, respectively. The results of the three most correlated pathways are shown in Figures 5A,B. The first three KEGG pathways and hallmark pathways were positively correlated with Linc00996. Genes related to Linc00996 were enriched in 77 KEGG pathways: allograft rejection, antigen processing and presentation, cell adhesion molecules (CAMs), autoimmune thyroid disease, and B-cell receptor signaling pathway, etc. Genes related to Linc00996 were enriched in 18 hallmark pathways including IL2-STAT5 signaling, IL6-JAK-STAT3 signaling, allograft rejection, complement, and inflammatory response.

### Immune infiltration analysis of Linc00996

The “gsva” R software package was applied to calculate the ratio of 24 immune cells in the standardized matrix of 331 cancer samples through “ssGSEA” algorithm, and the immune cells with significant differences were compared between high- and low-Linc00996 groups. Meanwhile, the diversities of immune cells between normal and tumor specimens were compared (*p* < 0.05). The boxplot exhibited the results through the “ggplot2” R package, as shown in Figure 5C. There were 23 types of immune cells with significant differences (*p* < 0.05): aDCs, B cells, CD8 T cells, cytotoxic cells, DCs, eosinophils, iDCs, macrophages, mast cells, neutrophils, NK CD56^bright^ cells, NK CD56^dim^ cells, NK cells, pDCs, T cells, T helper cells, Tcm cells, Tems, TFH cells, Tgd cells, Th1 cells, Th17 cells, and Tregs. The relevance between the proportion of immune cells with significant differences and model genes was calculated using the “psych” R package, and the threshold was *p* < 0.05. T cells, helper T cells, and cytotoxic cells were positively correlated with Linc00996 expression, cor >0.6 (Figure 5D).

### qPCR validation of Linc00996 in lung adenocarcinoma

To identify the results of bioinformatics analyses and the bearing between Linc00996 expression and clinicopathological features in LUAD, 61 freshly frozen lung cancer tissues and matched normal tissues were detected by qPCR. The expression level of Linc00996 was obviously connected with stages T2–4 and pathological stage. There was no significant association with age, region, gender, smoking history, and N stage (Table 2).

**TABLE 2:** Relationship between Linc00996 and the clinicopathological characteristics in 61 patients with LUAD.

Factor	Low Linc00996	High Linc00996	N	x^2^	*P*
All case	30	31	61	1.318	0.251
Age					
≤65	25	22	47		
>65	5	9	14		
Region					
Xuanwei	15	14	29	0.143	0.705
No-Xuanwei	15	17	32		
Gender					
Male	10	16	26	2.083	0.149
Female	20	15	35		
Smoking history					
Yes	7	11	18	1.082	0.298
No	23	20	43		
Tumor stage					
T1	25	18	43	4.68	0.031
T2–T4	5	13	18		
N stage					
N0	24	20	44	1.818	0.178
N1–3	6	11	17		
Pathological stage					
I–II	28	23	51	4.075	0.044
III–IV	2	8	10		

The *p-*value was measured by the chi-squared test or Fisher’s exact test.

### Linc00996 inhibits lung adenocarcinoma cell proliferation

**FIGURE 6:**
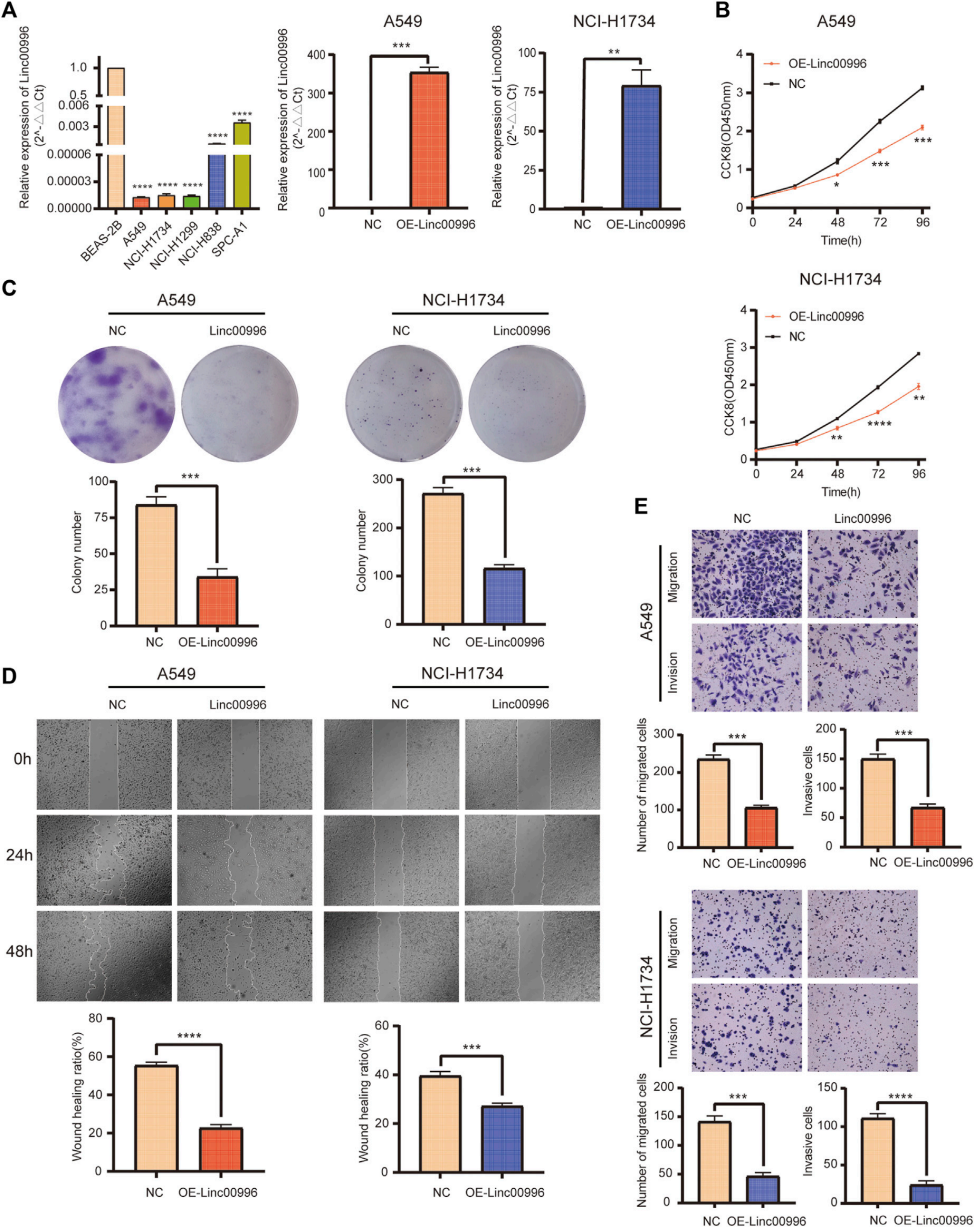
Linc00996 was critical for the cell growth, migration, and invasion of LUAD cells. **(A)** Linc00996 was underexpressed in LUAD cell lines (A549, NCI-H1734, NCI-H1299, NCI-H838, and SPC-A1). Overexpression of Linc00996 in A549 and NCI-H1734 cells was confirmed by RT-PCR. **(B)** Cell growth was inhibited by overexpression of Linc00996 in A549 and NCI-H1734 cells. **(C)** Overexpression of Linc00996 inhibited colony formation in A549 and NCI-H1734 cells. **(D)** Overexpression of Linc00996 inhibited cell migration, and wound healing assay was performed. **(E)** Overexpression of Linc00996 inhibited cell migration and invasion; representative images were shown. *p < 0.05, **p < 0.01, ***p < 0.001, ****p < 0.0001.

We established two stable overexpression Linc00996 LUAD cell lines (A549 and NCI-H1734). The effects of Linc00996 overexpression were verified by qPCR (Figure 6A). The ectopic expression of Linc00996 obviously suppressed the cell viability of A549 and NCI-H1734 (Figure 6B). Moreover, it was observed that the amount of colonies formed by A549 and NCI-H1734 cells markedly decreased compared to those formed by empty vector-transfected cells (Figure 6C). Hence, these results indicated that Linc00996 is indispensable for cell proliferation activity and ability in LUAD cells.

### Linc00996 suppresses lung adenocarcinoma cell migration and invasion

To investigate the motility and invasiveness of Linc00996 in LUAD cells, migration and invasion assays were carried out using transwell chambers, and monolayer wound healing assays were performed, respectively. Overexpression of Linc00996 markedly suppressed mobility and aggression in A549 cells and NCI-H1734 cells (Figure 6E). Monolayer wound healing assays revealed that ectopic-expressing Linc00996 had visibly reduced healing ability in LUAD cells after 2 days (22.75% of A549 cells and 27.29% of NCI-H1734 cells) (Figure 6D). Therefore, we suggested that aberrant expression of Linc00996 greatly enhances the invasiveness and migration ability in LUAD cells.

## Discussion

LncRNA was initially regarded as the noise of genome transcription and did not have biological functions (Liu et al., 2020). However, with the deepening of research and the progress of research methods, lncRNAs are considered to be involved in vital biological processes, including epigenetic regulation, chromatin modification, carcinogenicity, and transcriptional and post-transcriptional regulation (Shen and Zhou, 2020). Recently, aberrant expression of enormous amount of lncRNAs have been shown to get involved in many kinds of diseases, as well as malignant tumors, and a portion of lncRNAs have been found to regulate crucial signaling pathways related to tumorigenesis and development (Sun et al., 2018).

The coding region of Linc00996 is located on chromosome 7, and the length of mature Linc00996 is 2043 nt (Strausberg et al., 2002). Through a literature database search, Linc00996 was based on data mining methods using bioinformatics analysis, and Linc00996 was found to be a potential prognostic molecular marker in colorectal cancer, multiple myeloma, and HNSCC (Ge et al., 2018; Zhou et al., 2020; Lina, 2021). However, Linc00996 is rarely reported in lung cancer, and the molecular mechanism remains elusive.

Our survey systematically and comprehensively reviewed the clinical significance and prognosis of Linc00996 in LUAD. By mining TCGA and other databases, as well as our own experimental validation, we confirmed that Linc00996 is downregulated in cancer tissues and is a favorable prognostic factor in LUAD.

LUAD is one of the main pathological subtypes of NSCLC, and it is significantly different from lung squamous cell carcinoma in pathogenesis and clinical features (Pan et al., 2014; Faruki et al., 2017). In our research, only LUAD was selected as the focal point because LUAD accounts for an increasingly crucial proportion of lung cancer morbidity and mortality, and another vital cause was to avoid potential biases owing to diverse biological characteristics and molecular mechanisms. It is still indispensable to further discuss whether Linc00996 plays a key role in other pathological subtypes of lung cancer.

In TCGA-LUAD and GSE31210, Linc00996 expression in normal samples substantially exceeded compared to that in cancer samples. In TCGA-LUAD, Linc00996 had substantial differences in gender, different pathological stages, different T stages, and different N stages. There were significant differences in Linc00996 among different pathological stages in GSE37745. Univariate and multivariate Cox analyses were performed in TCGA-LUAD and GSE37745 to examine whether Linc00996 was an independent prognostic factor in patients with LUAD. In TCGA-LUAD, the three traits of N stage, T stage, and pathological stage were harmful factors. Linc00996 high-expression groups were protective factors. In GSE37745, Linc00996 was a beneficial factor; on the contrary, pathological stage was harmful. Interestingly, Linc00996 was also an independent predictor of prognosis in multiple myeloma patients in a study that established a survival model based on weighted gene co-expression network analysis (WGCNA) and principal component analysis (PCA) (Zhou et al., 2020). Previously, all studies about the expression level and clinical significance of Linc00996 in LUAD were found on bioinformatics analysis. Therefore, in order to identify the results of bioinformatics analyses, 61 freshly frozen lung cancer tissues and matched normal tissues were carefully analyzed using qPCR data. The expression level of Linc00996 was definitely linked with T stage and pathological stage. There was no significant association with age, region, gender, smoking history, and N stage. Although the verified results were not completely consistent with bioinformatics analyses, it was undeniable that Linc00996 was obviously downregulated in LUAD tissues, which has been confirmed to be tightly linked with the malignant phenotype. Linc00996 was obviously downregulated in LUAD tissues, and it had been confirmed that it is associated with malignant phenotypes. Taken together, these research studies illustrated that the deletion of Linc00996 was an indispensable key factor in tumorigenesis and progression in LUAD.

Tumor microenvironments (TMEs), interacting with tumor cells and mutually mediates the immunotolerance of malignant tumors, especially the immune microenvironment dominated by immune cells, play an essential role in the prognosis of various tumors (Zhang et al., 2018). A study proved that Linc00996 is co-expressed with 75 immune-related genes, far exceeding other lncRNAs. Meanwhile, sequencing of ectopic expression of Linc00996 LUAD cells identified that DEGs were actively engaged in immune-related pathways (Yan et al., 2021). Analyses of data from 331 cancer samples indicated that Linc00996 expression levels influence and modulate immune cell levels in the LUAD immune microenvironments. There was a significant relationship between Linc00996 expression and the levels of DCs, B cells, CD8 T cells, cytotoxic cells, eosinophils, macrophages, mast cells, neutrophils, NK cells, T cells, and T helper cells. Lung cancer patients with high levels of immune cell infiltration in TMEs might have a greater fortune of survival (Zhang et al., 2020). Linc00996 could affect the level of immune infiltration by mediating gene expression levels in these tumor-infiltrating immune cells and participating in immune-related signaling pathways.

A study in colorectal cancer showed that downregulation of Linc00996 expression is strongly linked with the tumorigenesis, metastasis, and poor prognosis, indicating that Linc00996 directly participates in or indirectly regulates pivotal genes in the JAK-STAT and PI3K-AKT and other signaling pathways, thereby affecting the tumorigenesis and critical transfer pathways of colorectal cancer (Ge et al., 2018). Interestingly, we also found that the antigen processing and presentation, JAK-STAT3, and cell adhesion molecular signaling pathways play important roles in LUAD, as shown by KEGG and hallmark pathways. They have been confirmed to be involved in tumorigenesis and disease progression (Rosenthal et al., 2019). Therefore, we speculated that Linc00996 inhibits the occurrence and development of LUAD by modulating these signaling pathways. A defective antigen presentation mechanism is one of the pathways of cancer immune escape (Jhunjhunwala et al., 2021). Studies have shown that NSCLC often has changes in human leukocyte antigen class I (HLA‐I) gene expression, which impairs antigen presentation and promotes immune escape (Perea et al., 2018; Castro et al., 2019). A study published in Cell believes that HLA LOH exists in 40% of NSCLC and explores the immune escape mechanism of lung cancer from this perspective (McGranahan et al., 2017). Other studies have shown that the higher the expression of tumor mutational burden and tumor neoantigen in the tumor tissue of NSCLC patients, the better the efficacy of anti-PD-1 therapy, and the longer the progression‐free survival of patients (Rizvi et al., 2015). The constitutional activation of STAT3 has been observed in bronchogenic carcinoma by researchers. Therefore, further study was carried out and found that more than 50% of NSCLC patients had abnormal activation of STAT3. Interestingly, in most NSCLC cells, abnormal activation of STAT3 was also discovered (Yu and Jove, 2004). Patients with high STAT3 expression have a worse tumor stage and prognosis (Xu and Lu, 2014; Wu et al., 2016; Tong et al., 2017). In addition, some studies have stated that reducing JAK/STAT activity can inhibit lung cancer metastasis (Lin et al., 2013; Chuang et al., 2017). The pathophysiology and mechanism of cell adhesion have been extensively reported, and it was involved in many diseases from developmental intellectual disability to malignant tumors. Indeed, two main features of malignancies, loss of adhesion between cells and anchorage-independent growth, are closely related to cell adhesion (Janiszewska et al., 2020). Nevertheless, the definite functions of Linc00996 in TMEs and molecular signaling pathway mechanisms still need more in-depth and multi-faceted research.

The invasion-metastatic cascade of cancer cells is a crucial factor that seriously affects the cure rate and survival time of tumor patients. Lung cancer patients with strong invasive and metastatic ability are prone to early metastasis and have a poor prognosis (Hashimoto et al., 2016; Alam et al., 2020). Therefore, we conducted a preliminary study on Linc00996. A suite of cell functional assays identified that Linc00996 has the capacity of inhibiting the invasion and metastasis cascade of tumor cells in LUAD. In *in vitro*, ectopic expression of Linc00996 remarkably suppressed cell growth viability and colony formation in two LUAD cell lines (A549 and NCI-H1734). The two experiments powerfully demonstrated that Linc00996 serves as a tumor silencer in LUAD. Meanwhile, the effect of Linc00996 on cell migration and invasion was also assessed. After transfection overexpressing Linc00996, cell motility and invasive capacity were significantly inhibited in two LUAD cell lines. The extracellular functional verification of Linc00996 is tightly linked to the molecular mechanism of cell adhesion, but the precise role of Linc00996 in the cell adhesion molecular mechanism requires further research.

## Conclusion

Based on the mining of TCGA and GSEA data, Linc00996 expression was significantly downregulated in LUAD, compared with adjacent normal lung tissues. Deletion of Linc00996 is inextricably linked with OS in LUAD patients. In addition, Linc00996 may repress the tumorigenesis and metastasis of LUAD via some tumor-related signaling pathways, such as antigen processing and presentation, JAK-STAT3, and cell adhesion. We performed some validations on LUAD tissues as well as a series of cell function experiments *in vitro*. However, further research studies are still requisite to corroborate and enrich our understanding of Linc00996 and its precise role in LUAD or other lung cancer pathological subtypes.

## Data Availability

The original contributions presented in the study are included in the article/Supplementary Material; further inquiries can be directed to the corresponding authors.
