# Supplementary material for: Linc00996 is a favorable prognostic factor in LUAD: Results from bioinformatics analysis and experimental validation
Source: Front Genet. 2022 Sep 2;13:932973. doi: 10.3389/fgene.2022.932973 (PMC9479463; doi:10.3389/fgene.2022.932973)
Supplement: Supplementary file 1 [file Table1.docx]

| Supplementary Table S1. The Primer sequence in research. | |
| --- | --- |
| Primer | Sequence（5'→3'） |
| LINC00996-F | CTCTGCCACATCGTTCGGTTC |
| LINC00996-R | CTTCTTACGCTGCCAACTGCTAA |
| GAPDH-F | GGAGCGAGATCCCTCCAAAAT |
| GAPDH-R | GGCTGTTGTCATACTTCTCATGG |
