# Supplementary material for: Linc00996 is a favorable prognostic factor in LUAD: Results from bioinformatics analysis and experimental validation
Source: Front Genet. 2022 Sep 2;13:932973. doi: 10.3389/fgene.2022.932973 (PMC9479463; doi:10.3389/fgene.2022.932973)
Supplement: Supplementary file 2 [file Table2.docx]

Carrier


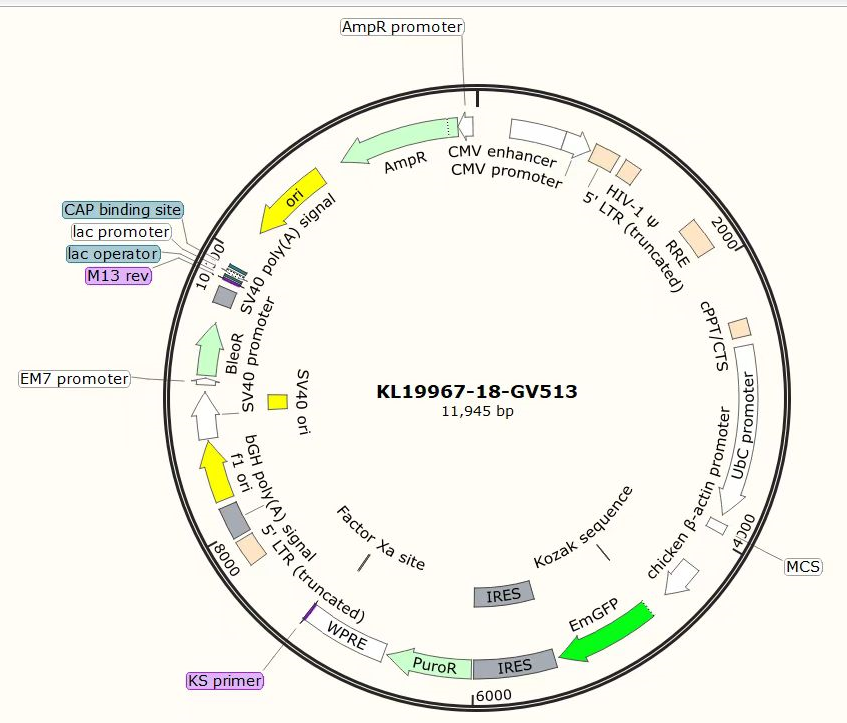


Sequencing Analysis

AATTGTCCGCTAAATTCTGGCCGTTTTTGGCTTTTTTGTTAGACGAAGCTTGGGCTGCAGGTCGACTCTAGAGGATCCGGAAGAAGTGTATTCAGCATTCTACCTTCTTCCTGATTCTGTGAGCTTAGACCTGCTTCCACTTTCAATCTCTCTCATTGCAGGCTGAAGAGGACTGGCCTTTCAGTGGCCCTCGTGGAACGGTAGGAAGCTGAGCTGGACTCTCTGCCACATCGTTCGGTTCCCACAACAGCCTGATGAAGCAGCAGCAGAAAAAATGGAAGCCATTTTGGTTAACGGGTCACATCTTAGCAGTTGGCAGCGTAAGAAGCGGAAGGATCCACTGGTCAGAAATGGAGATTGAGGAGAAGCTGAGAGCACGGAGGGCTTCAGGGGAGAGCCTGTCCTTTGGAAAAGCGGGCAATTTGCACTCGTGGTAGATGAGTGGGGAGGCCGCTCAGTCTCTCTGCCCCTTTGCACATGCTGCACCCGTTTAGTTTGACGTCGTGGAGAGCTCCTCCAGGCACGGGCTGTGTGAAAGGGTTTAAAAGTTTGGGGGCATGGCTGGGGGTCCCCGCATGAGGGGTGCTGTGTAGGCTCCCATCTTTTCTGCCGGTCATGCTGGGAGCCAACAGCACCGGCTCCTGACGTGGGGACTTGCCCTGTGCCATGACGTGGGGACTCGCCCTGTGCCCTGCGCCCCGGTTCCTGCTGGTTCTGTGCCTTCTGGACTCATTTCCTTTCCAGGGTCCGCAACCGCTTCCGACACAATCACTATTTTGGCTGCGGCCACGGCTTCATTCGCTTCAGCTGCGAGCTTTTCTCAGCGCAGAGAGCACAGGGAAGCTGGGAGGAGTCCGCCTAGATGGAATCTTCCAGAAGCAACATGACTCTTTTAGGCCTTTGCTGCCAGCTAAGAAGGTGAAGAAAAGTCACCACTGCAATAAAAAGAGGGCACTTTGTCTTACTTGGCAATAAACCACCACCAGCAGCAGCAGCAATAGCAGCACCCCCACCCCCACCAAACCCAAAACAAACAAGAAAAGCAAAAGGAAACCTTCGTTTTCAGTGAGAGGATTGGCATGAAGAATCCTTGGGACACAGGAATCAATCAAGACCTTTGGATGCCCCTGCCTGCTCTGTCTCTAGAAGCCTCAAGCCCAGCATCCCCATGCCGACGTGCTCATCTCTAGCAAAACACCAGCCTCTCAGCCCTCTCTCCATAAAGGCCACTAGAAAGTACTGTGATGCCAGGCTGCTCCACATCCAGGCAGCCTGGGTGACCACTGCCCTTTATGAGAATCAGAAAAAGCTGTCTCCCGAGTAGTTGGAATTACAGGTGCATGCCACCATGCCTGGCTAATTTTTGTATTTTTAGTAGAGATGGAGTTTCACTATGTTGGCCAGGCTGCTCTTGAACTCCTGACCTCAAATGATCCACCCGACTCGGCCTCCCAAAGAGCTGGAATTACAGGAGAAGCTAAACGTTTTAAAATAAGTATATTATTTCTGAATTACTATAACAGACATGGACTCCATTTGAACAAGATACTTAATTCCATCAAAAACTTGTCAAAATATACAAATTTAGTCACAAGACACTTTACTACCATCTACCAAAGCTGTAGTAAACACACACATTTTCCATCTTTGAAATGAAATAACGTATAATAATGTTTTCCAAAATAACAAAGATGTCTGCAGTGAATAGCACTCCCTTTGATGTGGCAACTGGGTCAATCGTGTTAACAACTGTGAATTCATCTTTTTAAGAAATTCTTTTATTGTTTCTTTTCTTTTCCGCGACAGTCTCACTCGTGTTGCCCCGGCTGGAGTGCAGTGGCGCTATCTTGGCTCACTGCAGCCTCCGCCTCCCGGGTTCAAGCAATTCTCATGCCTCAGCCTCCTGAGTAGCTGGGATTATAGGCACGTGCCACCACACCCAGCTAATTTTTTTGTATTTTTAGTAGAGACGGGGTTTGACCATGTTGGTCAGGGTGGTCTCGAATTCCTGACCTCAGTTGAGCCGCCGGCCTCGGCCTCCCAGGAAGTGCTGGGATTACAGGCATGAGCCACCAGGCCCGGCCTTATTGTTTCTATTTGTAATAAATCTGTTCCCCTTGCGCTAGCACATAACTTACGGTAAATGGCCCGCCTGGCTGACCGCCCAACGACCCCCGCCCATTGACGTCAATAGTAACGCCAATAGGGACTTTCCATTGACGTCAATGGGTGGAGTATTTACGGTAAACTGCCCACTTGGCAGTACATCAAGTGTA
