# Supplementary material for: Linc00996 is a favorable prognostic factor in LUAD: Results from bioinformatics analysis and experimental validation
Source: Front Genet. 2022 Sep 2;13:932973. doi: 10.3389/fgene.2022.932973 (PMC9479463; doi:10.3389/fgene.2022.932973)
Supplement: Supplementary file 3 [file Table3.docx]

Supplementary Table S3. The correlation coefficient R and statistical discrepancy of Figure 4B.

| gene | cor | p.value |
| --- | --- | --- |
| IGHM | 0.408573393 | 9.49E-15 |
| IGHJ3P | 0.551970952 | 8.68E-28 |
| IGKJ5 | 0.488122212 | 3.22E-21 |
| CXCL9 | 0.525765067 | 6.36E-25 |
| CCL19 | 0.607873135 | 8.02E-35 |
| CXCL13 | 0.524300447 | 9.04R-25 |
| SCGB1A1 | 0.303322657 | 1.79E-08 |
| SFTPC | 0.354020985 | 3.30E-11 |
| SFTPA1 | 0.307965731 | 1.03E-08 |
| SFTPA2 | 0.298059387 | 3.23E-08 |
